# Chinese neurologists’ perspective on intravenous thrombolysis for acute ischemic stroke

**DOI:** 10.1002/brb3.882

**Published:** 2017-12-07

**Authors:** Meng‐Die Wang, Xiao‐Xv Yin, Ting‐Ting Yang, Yong Wang, Yi‐Yi Zhu, Yan‐Feng Zhou, Zu‐Xun Lu, Bo Hu

**Affiliations:** ^1^ Department of Neurology Union Hospital Tongji Medical College Huazhong University of Science and Technology Wuhan China; ^2^ School of Public Health Tongji Medical College Huazhong University of Science and Technology Wuhan China; ^3^ The Stroke Quality Control Center of Hubei Province Wuhan Hubei China; ^4^ Key Laboratory of Neurological Disease Ministry of Education Tongji Medical College Huazhong University of Science and Technology Wuhan China

**Keywords:** acute ischemic stroke, China, neurologists, perspective, thrombolysis

## Abstract

**Objectives:**

This study examined the neurologists’ perspective toward intravenous thrombolysis for the treatment of acute ischemic stroke and the influencing factors in a Chinese Province.

**Methods:**

A cross‐sectional study was conducted from 1 October 2014 to 31 January 2015. A total of 719 neurologists from 66 hospitals in Hubei Province were included. A questionnaire was designed, and multivariable logistic regression models were used to identify the factors associated with the neurologists’ perspective toward intravenous thrombolysis.

**Results:**

Among the responding neurologists, 67.3% reported using intravenous thrombolysis and 32.9% believed the treatment was unsafe. Approximately 51.4% reported deficits in their knowledge of intravenous thrombolysis and 45.8% felt unconfident about their ability to employ the treatment. The majority (90.1%) supported hospitals in performing intravenous thrombolysis for eligible patients. Their safety concern was associated with hospital grade (odds ratio[OR] = 2.3; 95% confidence interval [CI], 1.4–3.7) and previous experiences with thrombolysis (OR = 3.1; 95% CI, 2.1–4.6). Their confidence was associated with their educational background (OR = 2.5; 95% CI, 1.3–4.5), knowledge mastery (OR = 10.4; 95% CI, 6.6–16.3), and previous experiences with thrombolysis (OR = 3.3; 95% CI, 2.1–5.3). Their attitudes were associated with gender (OR = 0.6; 95% CI, 0.3–1.0) and previous experiences with thrombolysis (OR = 4.9; 95% CI, 2.5–9.4).

**Conclusions:**

Most neurologists in Hubei Province, China, identified with intravenous thrombolysis for the treatment of acute ischemic stroke. However, they were weak in knowledge and lack confidence. Therefore, training, especially practical training, is needed to promote the use of thrombolysis in the region.

## 1 INTRODUCTION

Stroke has now become the leading cause of death and disability worldwide (Levine, 2015). Although great progress has been made in stroke treatment, prevention, and rehabilitation over the past decade, huge challenges remain. Intravenous (IV) recombinant tissue plasminogen activator (rt‐PA) is now a level‐1A treatment available for acute ischemic stroke (AIS) (Grotta, 2014). Since its approval by Food and Drug Administration (FDA) in 1996 (Adams et al., 1996), increasing AIS patients have benefited from the treatment. Despite its efficacy in treatment of AIS, to date, the thrombolysis rate remains extremely low. In China, only 2% of AIS patients received IV thrombolytics (Wang et al., 2011), which was lower than the rates in other countries (Adeoye, Hornung, Khatri, & Kleindorfer, 2011; Heuschmann et al., 2003; Sato et al., 2009).

A number of obstacles to the extensive use of IV thrombolysis have been identified, and patients’ delayed presentation is believed to be the most important constraint (Dirks & Dippel, 2013; Eissa, Krass, & Bajorek, 2012; Yin et al., 2016). However, we previously found 37.9% of the investigated AIS patients presented within 4 hr after symptom onset and had enough time to receive thrombolytic therapy (Zhou et al., 2016). According to a report, the thrombolysis rate in China was 2% (Wang et al., 2011). This means that more than 90% of the aforementioned patients presenting within 4 hr after onset might not receive thrombolysis. The data suggested that in‐hospital barriers should not be ignored and need further study. As core members of a stroke team, neurologists play important roles in the decision‐making regarding the treatment (Hovsepian & Karceski, 2013). Those who identify with the treatment are more inclined to recommend thrombolysis to their patients, whereas those who are relatively weak in the treatment tend to be hesitant or unwilling to use the treatment (Villar‐Cordova, Morgenstern, Barnholtz, Frankowski, & Grotta, 1998; Wang et al., 2011). Therefore, identifying the perspective of neurologists toward IV thrombolysis is necessary and will help promote the application of the treatment in eligible AIS patients.

However, only few studies investigated the use of thrombolysis from the perspective of physicians. All of them were qualitative studies and conducted in developed countries, such as the United States, Canada, and several European countries (Bobrow et al., 2009; Brown, Barsan, Lisabeth, Gallery, & Morgenstern, 2005; Katzan, Sila, & Furlan, 2001; Leira, Pary, Davis, Grimsman, & Adams, 2007; Mellon, Hasan, Lee, Williams, & Hickey, 2015; Shamy & Jaigobin, 2013; Villar‐Cordova et al., 1998). Therefore, we conducted a cross‐sectional survey among neurologists in Hubei Province in China to understand their perspective toward IV thrombolysis for the treatment of AIS and the influencing factors.

## 2 METHODS

### 2.1 Ethics statement

The study was performed in accordance with the principles of the Declaration of Helsinki and was approved by the Research Ethics Committee of Tongji Medical College, Huazhong University of Science and Technology, Wuhan, China. Written informed consents were obtained from each respondent. All the information provided by participants was kept confidential.

### 2.2 Setting and participants

**Figure 1:**
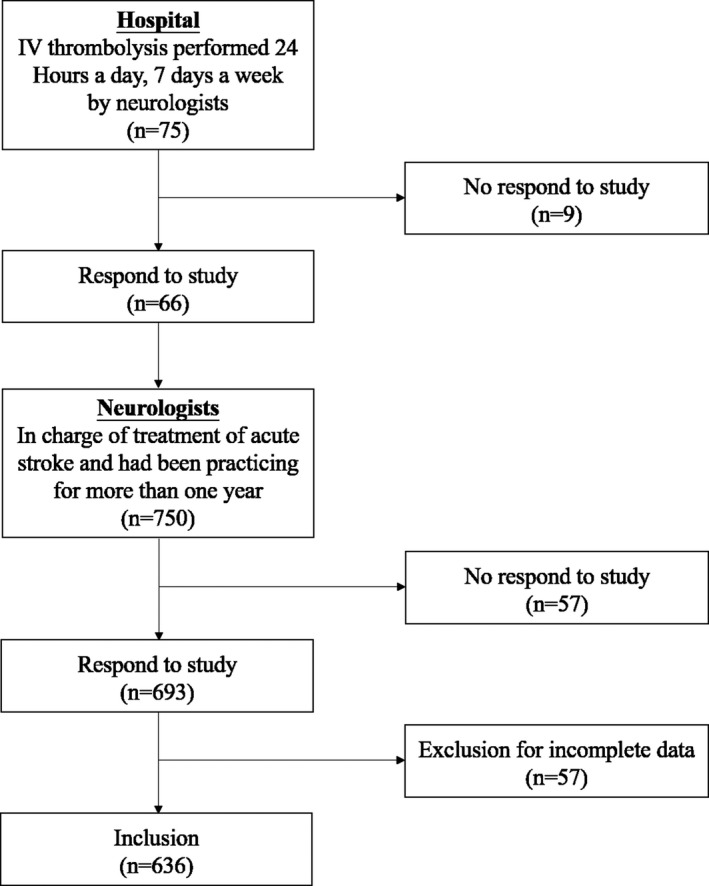
Selection process of eligible questionnaires. This cross‐sectional study was conducted in Hubei Province, and data were collected from 1 October 2014 to 31 January 2015. A total of 75 hospitals which have department of neurology and have the qualification to perform IV thrombolysis (24/7) were initially invited to join the project. All the neurologists from these hospitals were investigated if they had been in charge of treatment of acute stroke and had been practicing for more than one year. Eventually, 66 (88.0%) hospitals agreed to participate. We surveyed 750 neurologists in the participating hospitals and 693 (92.4%) neurologists responded to our questionnaire investigation. Fifty‐seven were excluded for incomplete data. Finally, 636 eligible questionnaires were included in the data analysis

This cross‐sectional study was conducted in Hubei Province, geographically located in central China, and data were collected from 1 October 2014 to 31 January 2015. A total of 75 hospitals which have department of neurology and have the qualification to perform IV thrombolysis were initially invited to join the project, including 38 grade III hospitals and 37 grade II hospitals. Moreover, IV thrombolysis was performed 24 hours a day, 7 days a week by neurologists in these hospitals. All the neurologists from these hospitals were investigated if they had been in charge of treatment of acute stroke and had been practicing for more than one year. Neurologists not involved in the treatment and those still on training programs were excluded (Figure 1).

### 2.3 Data collection

A standard structured questionnaire was designed on the basis of similar previous studies and by consulting specialists. The questionnaires were mailed to the subjects. All neurologists investigated were asked to finish the questionnaires on anonymous basis. The items of the questionnaire covered demographic data, experience with, knowledge of, and attitude toward the thrombolytic therapy. Demographic data included age, gender, education, job title, hospital grade, working duration in hospital. As to job title, clinicians in China fall into four major categories: resident, attending physician, associate chief physician, and chief physician, in terms of their competence levels and years of service. With respect to hospital grade, generally speaking, hospitals are rated according to their capacity and functions, and grade III hospital had better capacity than grade II hospital.

### 2.4 Statistical analysis

Statistical analysis was performed by employing Statistical Product and Service Solutions 12.0 for Windows. For descriptive analysis, frequency and percentage were used for independent variables. Multivariate logistic regression was used to identify the factors associated with the neurologists’ safety concern, confidence, and attitudes toward intravenous thrombolysis. Variables input into the model were believed to bear plausible association with the outcome and covered age, gender, education, job title, hospital grade, working duration in hospital, whether the respondent had mastered the knowledge of thrombolysis, and whether the respondent had previous experiences. Adjusted odds ratios (ORs) and 95% confidence intervals (CIs) for variables were obtained. For all analysis, differences were tested using two‐tailed tests, and a *p *<* *.05 was considered to be statistically significant.

## 3 RESULTS

Seventy‐five hospitals were invited to join the project initially. Eventually, 66 (88.0%) hospitals, including 34 grade III hospitals and 32 grade II hospitals, agreed to participate (Table S1). These hospitals were located in 13 cities across the province, each city having at least two hospitals involved in the study.

We surveyed 750 neurologists in the participating hospitals and 693 (92.4%) neurologists responded to our questionnaire investigation. Fifty‐seven were excluded for incomplete data. These neurologists just answer some questions about the demographic variables, and their experience with, knowledge of, and attitude toward the thrombolytic therapy were not filled in. Finally, 636 eligible questionnaires were included in the data analysis. The mean age of the subjects was 35.7 ± 7.7 years and 56.1% were male. The characteristics of the participants are shown in Table 1.

**Table 1:** Characteristics of responding neurologists

Characteristic	*n*	%
Age, Mean ± SD	35.7 ± 7.7	
Gender		
Male	357	56.1
Female	279	43.9
Education		
Bachelor degree or lower	315	49.5
Master degree	269	42.3
Doctorate degree	52	8.2
Job title		
Attending physician or lower	464	73.0
Associate chief physician	114	17.9
Chief physician	58	9.1
Hospital grade		
Grade II	166	26.1
Grade III	470	73.9
Working duration in hospital, Mean ± SD	10.1 ± 8.1	

As shown in Table 2, of the 636 respondents, 428 (67.3%) reported using IV thrombolysis for AIS, with a mean frequency of 10.8 times. Nearly half (51.4%; 327/636) reported deficits in their knowledge of IV thrombolysis. Up to a third (32.9%; 209/636) believed that IV thrombolysis was unsafe. Approximately 45.8% (291/636) felt unconfident about their ability to employ the treatment. The majority (90.1%; 573/636) supported hospitals in performing IV thrombolysis for eligible AIS patients, whereas 9.9% (63/636) opposed the treatment.

**Table 2:** Neurologists’ perspective concerning IV thrombolysis for the treatment of AIS

	*n*	%
Did you treat AIS patients with IV thrombolysis?		
No	208	32.7
Yes	428	67.3
Did you master the knowledge of IV thrombolysis for AIS?		
No	327	51.4
Yes	309	48.6
Do you think IV thrombolysis for AIS is safe?		
No	209	32.9
Yes	427	67.1
Do you feel confident about your ability to employ the treatment?		
No	291	45.8
Yes	345	54.3
Do you support hospitals in performing IV thrombolysis for AIS patients?		
No	63	9.9
Yes	573	90.1

Multivariate logistic regression analysis exhibited that the neurologists who worked in a grade III hospital (OR, 2.3; 95% CI, 1.4–3.7) and those who had previously used thrombolysis (OR, 3.1; 95% CI, 2.1–4.6) were more likely to believe the treatment was safe. The neurologists who had doctorate degree (OR, 2.5; 95% CI, 1.3–4.5), those who mastered the knowledge of thrombolysis (OR, 10.4; 95% CI, 6.6–16.3), and those who had previous thrombolysis experiences (OR, 3.3; 95% CI, 2.1–5.3) were more inclined to be confident in using the treatment. The neurologists who had previously treated patients with thrombolysis were more willing to perform the treatment (OR, 4.9; 95% CI, 2.5–9.4), whereas female neurologists were more reluctant to perform the treatment (OR, 0.6; 95% CI, 0.3–1.0). (Table 3)

**Table 3:** Multivariate logistic regression analysis: Factors influencing neurologists’ perspective toward treating AIS patients with thrombolysis

Characteristic	Safety concern	Confidence	Attitudes
Age, year (Ref*:<35)			
35–50	1.1 (0.6–1.9)	1.4 (0.9–2.4)	0.9 (0.4–2.4)
>50	1.0 (0.3–3.3)	3.8 (0.9–17.4)	0.7 (0.1–3.3)
Gender (Ref: Male)			
Female	0.8 (0.6–1.2)	0.7 (0.4–1.1)	**0.6 (0.3–1.0)** *
Education (Ref: Bachelor degree or lower)			
Master degree	1.5 (0.9–2.4)	1.6 (1.0–2.8)	1.6 (0.8–3.4)
Doctorate degree	1.8 (0.8–4.3)	**2.5 (1.3–4.5)** *	2.0 (0.4–9.3)
Job title (Ref: Attending physician or lower)			
Associate chief physician	1.6 (0.8–3.1)	1.8 (0.8–3.8)	1.0 (0.4–2.8)
Chief physician	1.0 (0.4–2.7)	1.8 (0.5–6.3)	0.4 (0.1–1.5)
Hospital grade (Ref: Grade II)			
Grade III	**2.3 (1.4–3.7)** **	1.0 (0.5–1.7)	1.3 (0.7–2.8)
Working duration in hospital, year (Ref:<10)			
10–25	0.9 (0.5–1.6)	1.6 (0.9–3.1)	1.0 (0.4–2.6)
>25	1.2 (0.4–3.0)	2.2 (0.7–6.5)	1.0 (0.2–4.1)
Knowledge mastery (Ref: No)			
Yes	1.5 (1.0–2.2)	**10.4 (6.6–16.3)** ***	1.4 (0.7–2.7)
Previous thrombolysis experience (Ref: No)			
Yes	**3.1 (2.1–4.6)** ***	**3.3 (2.1–5.3)** ***	**4.9 (2.5–9.4)** ***

Ref* is reference. *p <* *.05; **p <* *.01; ***p <* *.001. Bold values mean data is significant.

## 4 DISCUSSION

In this study, for the first time, we assessed perspective of neurologists regarding IV thrombolysis for the treatment of AIS in Hubei Province, China, and found that 90.1% of responding neurologists in this study supported hospitals in performing IV thrombolysis for eligible AIS patients. The ratio was substantially higher than those of other similar studies. For instance, Villar‐Cordova et al. (1998) found that 60% of the American neurologists who had not treated patients with rt‐PA would do it if a suitable candidate presented. Another study conducted in the USA indicated that only 31.5% of non‐neurologist physicians in the survey were willing to give rt‐PA to an eligible AIS patient (Leira et al., 2007). It was also reported that 60% of American emergency physicians were likely to use rt‐PA (Brown et al., 2005). This increased acceptance might be ascribed to the fact that those studies were conducted much earlier. In recent years, with mounting evidence supporting the validity of thrombolytic therapy, the attitude of physicians toward the treatment is changing (Fugate & Rabinstein, 2014; Hacke et al., 2008; Jauch et al., 2013; Prabhakaran, Ruff, & Bernstein, 2015). In spite of the willingness to use the method, nearly half of the responding neurologists reported deficits in their knowledge and felt unconfident about their ability to employ the treatment. Up to a third of them believed that IV thrombolysis was unsafe and only 65.6% reported using IV thrombolysis for AIS.

Full mastery of the knowledge on thrombolysis is essential to the application of thrombolysis. Previous studies found that physicians working in teaching hospitals were reported to be more comfortable with use of thrombolysis compared to those practicing in nonteaching hospitals as physicians in teaching hospitals were more exposed to or kept in breast with new developments in thrombolysis in AIS (Moradiya, Crystal, Valsamis, & Levine, 2013; Schumacher et al., 2007). In this study, we also showed increased confidence of neurologists who mastered the knowledge of thrombolytic therapy. Hence, in view of substantial lack in the knowledge about thrombolysis, we believe it is necessary to strengthen training in this regard among neurologists.

An important finding of this study was that thrombolysis experiences of neurologists were independently associated with their perspective regarding the treatment. We found the respondents who had previously used IV thrombolysis were more likely to believe the treatment was safe, felt confident about their ability, and were willing to perform the treatment. Consistent with our results, findings by Brown et al. (2005) also suggested that physicians with previous thrombolysis experiences preferred to treat AIS patients with rt‐PA. We believe previous experiences might help them build up skills and became more experienced with the treatment, including selecting right patients and preventing thrombolysis‐related complications. The findings suggested that, in the training, effort should be devoted more to practical skills than to theoretical knowledge and only improvement in treatment skills increases the confidence of neurologists in thrombolytic therapy.

In this study, we first explored the neurologists’ perspective toward IV thrombolysis for the treatment of AIS in China. The results of this survey suggest that most neurologists in Hubei Province, China, identified with the IV thrombolysis for the treatment of AIS. However, they were weak in knowledge and lacked confidence in using the treatment. Therefore, stepping‐up training and attaching importance to improvement in practical skills are important for the wide application of thrombolysis in the region.

A few limitations of the current study need to be stated. First, neurologists’ knowledge of the thrombolytic therapy was subjective self‐evaluation rather than objective measuring based on questions about knowledge of the thrombolytic therapy, which may overestimate the percentage of neurologists with knowledge about thrombolytic therapy. Second, our sample was confined to the neurologists in a central province of China, the generalizability of the finding to neurologists in other districts of China is unclear.

## CONFLICTS OF INTEREST

All authors declare no conflicts of interest.

## References

[brb3882-bib-0001] Adams Jr, H. P. , Brott, T. G. , Furlan, A. J. , Gomez, C. R. , Grotta, J. , Helgason, C. M. , … Thies, W. (1996). Guidelines for thrombolytic therapy for acute stroke: a supplement to the guidelines for the management of patients with acute ischemic stroke. A statement for healthcare professionals from a Special Writing Group of the Stroke Council, American Heart Association. Stroke, 27, 1711–1718. 8784157

[brb3882-bib-0002] Adeoye, O. , Hornung, R. , Khatri, P. , & Kleindorfer, D. (2011). Recombinant tissue‐type plasminogen activator use for ischemic stroke in the United States: a doubling of treatment rates over the course of 5 years. Stroke, 42, 1952–1955. 21636813 10.1161/STROKEAHA.110.612358 PMC4114342

[brb3882-bib-0003] Bobrow, B. J. , Demaerschalk, B. M. , Wood, J. P. , Villarin, A. , Clark, L. , & Jennings, A. (2009). Views of emergency physicians on thrombolysis for acute ischemic stroke. Journal of Brain Disease, 1, 29–37. 23818807 10.4137/jcnsd.s2231 PMC3676339

[brb3882-bib-0004] Brown, D. L. , Barsan, W. G. , Lisabeth, L. D. , Gallery, M. E. , & Morgenstern, L. B. (2005). Survey of emergency physicians about recombinant tissue plasminogen activator for acute ischemic stroke. Annals of Emergency Medicine, 46, 56–60. 15988427 10.1016/j.annemergmed.2004.12.025

[brb3882-bib-0005] Dirks, M. , & Dippel, D. W. (2013). Implementation of thrombolysis for ischaemic stroke. Lancet Neurology, 12, 120–121. 23260190 10.1016/S1474-4422(12)70304-6

[brb3882-bib-0006] Eissa, A. , Krass, I. , & Bajorek, B. V. (2012). Barriers to the utilization of thrombolysis for acute cischaemic stroke. Journal of Clinical Pharmacy and Therapeutics, 37, 399–409. 22384796 10.1111/j.1365-2710.2011.01329.x

[brb3882-bib-0007] Fugate, J. E. , & Rabinstein, A. A. (2014). Update on intravenous recombinant tissue plasminogen activator for acute ischemic stroke. Mayo Clinic Proceedings, 89, 960–972. 24775222 10.1016/j.mayocp.2014.03.001

[brb3882-bib-0008] Grotta, J. C. (2014). tPA for stroke: important progress in achieving faster treatment. JAMA, 311, 1615–1617. 24756509 10.1001/jama.2014.3322

[brb3882-bib-0009] Hacke, W. , Kaste, M. , Bluhmki, E. , Brozman, M. , Dávalos, A. , Guidetti, D. , … ECASS Investigators (2008). Thrombolysis with alteplase 3 to 4.5 hours after acute ischemic stroke. New England Journal of Medicine, 359, 1317–1329. 18815396 10.1056/NEJMoa0804656

[brb3882-bib-0010] Heuschmann, P. U. , Berger, K. , Misselwitz, B. , Hermanek, P. , Leffmann, C. , Adelmann, M. , … German Stroke Registers Study Group; Competence Net Stroke (2003). Frequency of thrombolytic therapy in patients with acute ischemic stroke and the risk of in‐hospital mortality: the German Stroke Registers Study Group. Stroke, 34, 1106–1113. 10.1161/01.STR.0000065198.80347.C5 12663875

[brb3882-bib-0011] Hovsepian, D. , & Karceski, S. (2013). Stroke, tPA, and physician decision‐making. Neurology, 81, e102–e105. 24062347 10.1212/WNL.0b013e3182a94f3c

[brb3882-bib-0012] Jauch, E. C. , Saver, J. L. , Adams, H. P. , Bruno, A. , Demaerschalk, B. M. , Khatri, P. , … Summers, D. R. (2013). Guidelines for the early management of patients with acute ischemic stroke: a guideline for healthcare professionals from the American Heart Association/American Stroke Association. Stroke, 44, 870–947. 23370205 10.1161/STR.0b013e318284056a

[brb3882-bib-0013] Katzan, I. L. , Sila, C. A. , & Furlan, A. J. (2001). Community use of intravenous tissue plasminogen activator for acute stroke: results of the brain matters stroke management survey. Stroke, 32, 861–865. 11283383 10.1161/01.str.32.4.861

[brb3882-bib-0014] Leira, E. C. , Pary, J. K. , Davis, P. H. , Grimsman, K. J. , & Adams, H. P. Jr (2007). Slow progressive acceptance of intravenous thrombolysis for patients with stroke by rural primary care physicians. Archives of Neurology, 64, 518–521. 17420312 10.1001/archneur.64.4.518

[brb3882-bib-0015] Levine, S. R. (2015). Covering all the bases to improve acute stroke care. Lancet Neurology, 14, 25–27. 25435130 10.1016/S1474-4422(14)70302-3

[brb3882-bib-0016] Mellon, L. , Hasan, H. , Lee, S. , Williams, D. , & Hickey, A. (2015). Knowledge of thrombolytic therapy amongst hospital staff: preliminary results and treatment implications. Stroke, 46, 3551–3553. 26470774 10.1161/STROKEAHA.115.010327

[brb3882-bib-0017] Moradiya, Y. , Crystal, H. , Valsamis, H. , & Levine, S. R. (2013). Thrombolytic utilization for ischemic stroke in US hospitals with neurology residency program. Neurology, 81, 1986–1995. 24186911 10.1212/01.wnl.0000436946.08647.b5 PMC3854832

[brb3882-bib-0018] Prabhakaran, S. , Ruff, I. , & Bernstein, R. A. (2015). Acute stroke intervention: a systematic review. JAMA, 313, 1451–1462. 25871671 10.1001/jama.2015.3058

[brb3882-bib-0019] Sato, S. , Uehara, T. , Toyoda, K. , Yasui, N. , Hata, T. , Ueda, T. , … Minematsu, K. (2009). Impact of the approval of intravenous recombinant tissue plasminogen activator therapy on the processes of acute stroke management in Japan: the Stroke Unit Multicenter Observational (SUMO) Study. Stroke, 40, 30–34. 18948604 10.1161/STROKEAHA.108.524942

[brb3882-bib-0020] Schumacher, H. C. , Bateman, B. T. , Boden‐Albala, B. , Berman, M. F. , Mohr, J. P. , Sacco, R. L. , & Pile‐Spellman, J. (2007). Use of thrombolysis in acute ischemic stroke: analysis of the Nationwide Inpatient Sample 1999 to 2004. Annals of Emergency Medicine, 50, 99–107. 17478010 10.1016/j.annemergmed.2007.01.021

[brb3882-bib-0021] Shamy, M. C. , & Jaigobin, C. S. (2013). The complexities of acute stroke decision‐making: a survey of neurologists. Neurology, 81, 1130–1133. 23946306 10.1212/WNL.0b013e3182a55ec7

[brb3882-bib-0022] Villar‐Cordova, C. , Morgenstern, L. B. , Barnholtz, J. S. , Frankowski, R. F. , & Grotta, J. C. (1998). Neurologists’ attitudes regarding rt‐PA for acute ischemic stroke. Neurology, 50, 1491–1494. 9596018 10.1212/wnl.50.5.1491

[brb3882-bib-0023] Wang, Y. , Liao, X. , Zhao, X. , Wang, D. Z. , Wang, C. , Nguyen‐Huynh, M. N. , & Li, H. (2011). Using recombinant tissue plasminogen activator to treat acute ischemic stroke in China: analysis of the results from the Chinese National Stroke Registry (CNSR). Stroke, 42, 1658–1664. 21512182 10.1161/STROKEAHA.110.604249

[brb3882-bib-0024] Yin, X. , Yang, T. , Gong, Y. , Zhou, Y. , Li, W. , Song, X. , & Lu, Z. (2016). Determinants of emergency medical services utilization among acute ischemic stroke patients in Hubei Province in China. Stroke, 47, 891–894. 26768208 10.1161/STROKEAHA.115.011877

[brb3882-bib-0025] Zhou, Y. , Yang, T. , Gong, Y. , Li, W. , Chen, Y. , Li, J. , … Lu, Z. (2016). Pre‐hospital delay after acute ischemic stroke in Central Urban China: prevalence and risk factors. Molecular Neurobiology, 54, 3007–3016. 27032390 10.1007/s12035-016-9750-4

