# Supplementary material for: Chinese neurologists’ perspective on intravenous thrombolysis for acute ischemic stroke
Source: Brain Behav. 2017 Dec 7;8(1):e00882. doi: 10.1002/brb3.882 (PMC5853636; doi:10.1002/brb3.882)
Supplement: Supplementary file 1 [file BRB3-8-e00882-s001.docx]

**Supplement Table 1**

| **Number** | **Institution** |
| --- | --- |
| 1 | Department of Neurology, Union Hospital, Huazhong University of Science and Technology, Wuhan, Hubei, P. R. China |
| 2 | Department of Neurology, Wuhan Puren Hospital, Wuhan, Hubei, P. R. China. |
| 3 | Department of Neurology, Wuhan Puai Hospital, Huazhong University of Science and Technology, Wuhan, Hubei, P. R. China. |
| 4 | Department of Neurology, The Fifth Hospital of Wuhan, Jianghan University, Wuhan, Hubei, P. R. China. |
| 5 | Department of Neurology, Wuhan Integrated Tcm & Western Medicine Hospital (the First Hospital of Wuhan), Wuhan, Hubei, P. R. China. |
| 6 | Department of Neurology, the Central Hospital of Wuhan, Huazhong University of Science and Technology, Wuhan, Hubei, P. R. China. |
| 7 | Department of Neurology, Zhongnan Hospital of Wuhan University, Wuhan, Hubei, P. R. China. |
| 8 | Department of Neurology, Wuhan General Hospital of Guangzhou Military, Wuhan, Hubei, P. R. China. |
| 9 | Department of Neurology, Hubei Xinhua hospital, Wuhan, Hubei, P. R. China. |
| 10 | Department of Neurology, PLA 161th Hospital, Wuhan, Hubei, P. R. China. |
| 11 | Department of Neurology, General Hospital of the Yangtze River Shipping, Wuhan, Hubei, P. R. China. |
| 12 | Department of Neurology, Hubei Provincial Crops Hospital of Chinese People’s Armed Police Force , Wuhan, Hubei, P. R. China. |
| 13 | Department of Neurology, General Hospital of Wuhan Iron and Steel Corporation, Wuhan, Hubei, P. R. China. |
| 14 | Department of Neurology, the Central Hospital of Enshi Autonomous Prefecture, Enshi, Hubei, P. R. China. |
| 15 | Department of Neurology, Renmin Hospital of Wuhan University, Wuhan, Hubei, P. R. China. |
| 16 | Department of Neurology, Taihe Hospital, Hubei Medical University, Shiyan, Hubei, P. R. China. |
| 17 | Department of Neurology, the Central Hospital of Jingzhou, Jingzhou, Hubei, P. R. China. |
| 18 | Department of Neurology, the Central Hospital of Ezhou, Ezhou, Hubei, P. R. China. |
| 19 | Department of Neurology, the First People′s Hospital of Jingzhou, Jingzhou, Hubei, P. R. China. |
| 20 | Department of Neurology, the Central Hospital of Xiaogan, Xiaogan, Hubei, P. R. China. |
| 21 | Department of Neurology, the Central Hospital of Xiangfan, Xiangfan, Hubei, P. R. China. |
| 22 | Department of Neurology, the Central People’s Hospital of Yichang, First College of Clinical Medical Science, Yichang, Hubei, P. R. China. |
| 23 | Department of Neurology, the First People′s Hospital of Xiangfan, Xiangfan, Hubei, P. R. China. |
| 24 | Department of Neurology, the Central Hospital of Suizhou, Suizhou, Hubei, P. R. China. |
| 25 | Department of Neurology, the Hospital Affiliated to Medical School of Hubei University for Nationalities, Enshi, Hubei, P. R. China. |
| 26 | Department of Neurology, the First People′s Hospital of Jingmen, Jingmen, Hubei, P. R. China |
| 27 | Department of Neurology, the First People′s Hospital of Yichang, Yichang, Hubei, P. R. China. |
| 28 | Department of Neurology, the First People′s Hospital of Zaoyang, Zaoyang, Hubei, P. R. China. |
| 29 | Department of Neurology, the People′s Hospital of Hong’an, Hong’an, Hubei, P. R. China. |
| 30 | Department of Neurology, Zhongshan Hospital, Wuhan, Hubei, P. R. China. |
| 31 | Department of Neurology, the Ninth People’s Hospital, Wuhan, Hubei, P. R. China. |
| 32 | Department of Neurology, the People′s Hospital of Yingcheng, Yingcheng, Hubei, P. R. China. |
| 33 | Department of Neurology, the People′s Hospital of Yangxin, Yangxin, Hubei, P. R. China. |
| 34 | Department of Neurology, the People′s Hospital of Dangyang, Dangyang, Hubei, P. R. China. |
| 35 | Department of Neurology, the People′s Hospital of Zhongxiang, Zhongxiang, Hubei, P. R. China. |
| 36 | Department of Neurology, the People′s Hospital of Xishui, Xishui, Hubei, P. R. China. |
| 37 | Department of Neurology, the People′s Hospital of Honghu, Honghu, Hubei, P. R. China. |
| 38 | Department of Neurology, the People′s Hospital of Yicheng, Yicheng, Hubei, P. R. China. |
| 39 | Department of Neurology, the People′s Hospital of Qianjiang, Qianjiang, Hubei, P. R. China. |
| 40 | Department of Neurology, the People′s Hospital of Jingshan, Jingshan, Hubei, P. R. China. |
| 41 | Department of Neurology, the People′s Hospital of Jianli, Jianli, Hubei, P. R. China. |
| 42 | Department of Neurology, the Second People′s Hospital of Yichang, Yichang, Hubei, P. R. China. |
| 43 | Department of Neurology, the People′s Hospital of Tongshan, Tongshan, Hubei, P. R. China. |
| 44 | Department of Neurology, the People′s Hospital of Yicheng, Yicheng, Hubei, P. R. China. |
| 45 | Department of Neurology, the People′s Hospital of Hanchuan, Hanchuan, Hubei, P. R. China. |
| 46 | Department of Neurology, the People′s Hospital of Zhijiang, Zhijiang, Hubei, P. R. China. |
| 47 | Department of Neurology, the People′s Hospital of Macheng, Macheng, Hubei, P. R. China. |
| 48 | Department of Neurology, the People′s Hospital of Zigui, Zigui, Hubei, P. R. China. |
| 49 | Department of Neurology, Wuhan Jiangxia District First People’s Hospital, Wuhan, Hubei, P. R. China. |
| 50 | Department of Neurology, Zhongxiang Hospital of Traditional Chinese Medicine, Zhongxiang, Hubei, P. R. China. |
| 51 | Department of Neurology, Jingmen Rehabilitation Hospital, Jingmen, Hubei, P. R. China. |
| 52 | Department of Neurology, the People′s Hospital of Luotian, Luotian, Hubei, P. R. China. |
| 53 | Department of Neurology, the First Hospital of Laohekou, Laohekou, Hubei, P. R. China. |
| 54 | Department of Neurology, the Traditional Chinese Medicine Hospital of Jingmen, Jingmen, Hubei, P. R. China. |
| 55 | Department of Neurology, the Central Hospital of Huanggang, Huanggang, Hubei, P. R. China. |
| 56 | Department of Neurology, the People′s Hospital of Nanzhang, Nanzhang, Hubei, P. R. China. |
| 57 | Department of Neurology, the Central Hospital of Tianmen, Tianmen, Hubei, P. R. China. |
| 58 | Department of Neurology, Liyuan Hospital, Huazhong University of Science and Technology, Wuhan, Hubei, P. R. China. |
| 59 | Department of Neurology, the Sixth Hospital of Wuhan, Jianghan University, Wuhan, Hubei, P. R. China. |
| 60 | Department of Neurology, the Second Worker Hospital of Wuhan Iron and Steel Corporation, Wuhan, Hubei, P. R. China. |
| 61 | Department of Neurology, Tianyou hospital, Wuhan university of Science and Technology, Wuhan, Hubei, P. R. China. |
| 62 | Department of Neurology, the Third Hospital of Wuhan, Wuhan, Hubei, P. R. China. |
| 63 | Department of Neurology, the First Hospital of Danjiangkou, Danjiangkou, Hubei, P. R. China. |
| 64 | Department of Neurology, the Renhe Hospital of the Three Gorges University, Yichang, Hubei, P. R. China. |
| 65 | Department of Neurology, the People′s Hospital of Shiyan, Shiyan, Hubei, P. R. China. |
| 66 | Department of Neurology, the Second People’s Hospital of JingZhou, JingZhou, Hubei, P. R. China. |
